# Comparative evaluation of shaping ability of two nickel-titanium rotary systems using cone beam computed tomography

**DOI:** 10.1186/s12903-015-0019-5

**Published:** 2015-03-10

**Authors:** Berkan Celikten, Ceren Feriha Uzuntas, Sebnem Kursun, Ayse Isil Orhan, Pelin Tufenkci, Kaan Orhan, Kemal Özgür Demiralp

**Affiliations:** Faculty of Dentistry, Department of Endodontics, Ankara University, Besevler, 06560 Ankara, Turkey; Faculty of Dentistry, Department of DentoMaxillofacial Radiology, Ankara University, Besevler, 06560 Ankara, Turkey; Division of Pediatric Dentistry, Ministry of Health, 75th Year Ankara Oral and Dental Health Centre, Cebeci, 06590 Ankara, Turkey; Ministry of Health, Public Hospitals Agency of Turkey, Bahcelievler, 06490 Ankara, Turkey

**Keywords:** CBCT, New one shape, ProTaper next, Transportation, Volumetric changes

## Abstract

**Background:**

We evaluated and compared the effects of different NiTi rotary systems – ProTaper Next and New One Shape – on the volume of dentin removed, canal transportation, and canal curvature in extracted human teeth using CBCT scanning with different voxel sizes.

**Methods:**

Fifty extracted human maxillary first molars with mesiobuccal canal curvature (25-35°) were used. Specimens were instrumented with the ProTaper Next or New One Shape. Pre- and post-instrumentation scans were performed to compare transportation at the levels of 2, 5, and 8 mm and volumes with two different voxel sizes (0.125-and 0.100-mm^3^) using 3D CBCT images. This study evaluated and compare the volume of dentin removed, canal transportation, and canal curvature. Differences according to instrumentation and voxel sizes were assessed using the Mann–Whitney *U*-test and the Wilcoxon signed-rank test.

**Results:**

Significant differences were found between apical and coronal levels for both systems (p < 0.05) in canal transportation. In comparing the systems, similar values were found at each level, without significant difference (p > 0.05) in terms of canal curvature and volume. Voxel sizes did not affect the measurements on canal volume, curvature or transportation; no significant difference was found between the 0.100- and 0.125-mm^3^ voxel sizes (p > 0.05).

**Conclusions:**

Both instrumentation systems produced similar canal transportation and volume changes. The two voxel resolutions also showed similar results, however a 0.125-mm^3^ voxel size can be recommend for a flat panel CBCT scanner with lower exposure dose.

## Background

Conventional endodontic treatment is based on shaping, disinfecting, and filling the root canal system [1]. A prepared root canal should have a continuously tapered funnel shape, while maintaining the original outline form of the canal [2]. However, these objectives are often difficult to achieve because of the highly variable root canal anatomy and canal curvature [3].

Several enlargement techniques have been developed to minimize errors, such as ledging, zipping, loss of working length, and apical transportation [4]. Although various root canal preparation techniques have been developed to overcome the problems, rotary nickel-titanium (NiTi) systems were developed to maintain the original canal shape and thus remain better centered [5-8].

ProTaper Next (Dentsply Maillefer, Ballaigues, Switzerland) is a novel system designed with variable tapers and an off-centered rectangular cross section. The set includes five shaping instruments with overall variable tapers [9]. Such a single-length technique possibly requires greater torsional strength resulting in higher stresses over its entire length [10]. These instruments are manufactured from so-called M-Wire raw material, which was shown to possibly extend fatigue life beyond that of conventional NiTi alloy [11].

Recently, a new concept in root canal preparation has been introduced with the New One Shape (Micro Mega, Besancon Cedex, France), which is claimed to complete canal shaping with only a single file in continuous rotation. The One Shape file is a single system that presents a variable asymmetrical cross-sectional geometry along the blade [12]. These instruments are also manufactured from M-Wire raw material [11]. The manufacturer claims that this particular instrument geometry facilitates canal preparation and the upward removal of debris.

Image quality has been described as the visibility of diagnostically important structures in the computed tomography images [13,14]. Voxel size has been reported to have a positive correlation with image quality (*e.g.*, contrast and resolution), as well as exposure dose [15,16]. The use of cone-beam computed tomography (CBCT), and particularly systems that provide a limited field of view image at low doses with sufficient spatial resolution, are recommended for applications in endodontic diagnosis, treatment planning, and post-treatment evaluation [17]. To date, a few studies have assessed the influence of voxel size on the diagnostic ability of a CBCT unit in evaluating root canal anatomy and also pathologies, such as simulated vertical/horizontal root fractures [18-20]. Recent studies showed that the visibility of the root canal anatomy could vary with respect to the specific protocol chosen to create the scan and reconstruct the images [21]. Although it was believed that images with a lower slice thickness and smaller voxel size would provide more and better information – and higher accuracy was reported with smaller voxel sizes [18-21]. There is no objective evidence for this, particularly before and after the preparations of root canals.

To our knowledge, few reported studies have compared the newly developed rotary systems [12,22-24]. However, no reported study has yet compared the “ProTaper Next” and “New One Shape” systems with various voxel sizes using CBCT. Thus, the aim of this study was to compare the effects of two NiTi rotary systems – ProTaper Next and New One Shape – on the volume of dentin removed, canal transportation, and canal curvature in extracted human teeth using CBCT scanning with different voxel sizes.

## Methods

Fifty extracted human maxillary first molars with two separate mesial canals and intact, mature root apices were included in the study. The teeth were selected on the basis of their similar characteristics in terms of length (20–22 mm) and mesiobuccal canal curvature (25-35°). Mesiobuccal root canals of maxillary molars were used in this study because they usually have severely curved canals.

Teeth were accessed using an EndoAccess bur (Dentsply Maillefer) under continuous water-cooling, and the mesiobuccal canals were localized and explored with a size 10 K-file (Dentsply Maillefer). Determination of the working length was performed at × 8 magnification using a surgical microscope (Opmi-Pico; Karl Zeiss, Jena, Germany) by inserting a #10 K-file to the root canal terminus and subtracting 1 mm from this measurement.

Specimens were divided randomly into two experimental groups (*n* = 25) according to the rotary NiTi file system used in canal instrumentation, the ProTaper Next (Dentsply Maillefer) or the New One Shape (Micro-Mega). Root canal instrumentation was performed by a single operator in accordance with the manufacturers’ instructions. Preparations were performed from the crown to the root apex of each tooth. To achieve uniform master apical size, the final apical preparation was set to #25 in each group. All canals were instrumented with hand pieces powered by a torque control motor (X-Smart; Dentsply Tulsa Dental, Tulsa, OK).

In the ProTaper Next group, the ProTaper Universal SX was used to enlarge the coronal aspect of the canal at a rotational speed of 300 rpm with a torque of 4 Ncm. This was followed by using the ×1 to working length, and canal finishing was performed with the *×*2 to working length.

In the New One Shape group, the Endoflare was used to 3 mm depth to enlarge the coronal aspect of the canal, followed by G1 and G2, which were used to the working length at 400 rpm with a torque of 2,5 Ncm (taper 25/0.06). The canal-shaping procedure was finished in three steps with the New One Shape instrument.

At the end of the root canal preparation, one tooth from the ProTaper Next and three from the New One Shape group were excluded from the study because of apical fractures during root canal treatments. Thus, the total numbers were finally 24 in the ProTaper Next and 22 in the New One Shape groups.

Irrigation was performed in each group with 2 mL of 5.25% NaOCl after the use of each file and when root canal instrumentation was complete. The smear layer was removed in all teeth using 1 mL of 17% ethylenediamine tetraacetic acid for 1 min, followed by a final flush with 5 mL of NaOCl. All rotary instruments used were discarded after one use to prevent file breakage.

### Scanning protocol

The teeth were coded and a 1.5-cm Plexiglas sphere was used to simulate the soft tissue. The teeth were placed into Plexiglas sphere one by one with wax from the root to an upright position. The Plexiglas was then mounted horizontally to fit the chin support of the machine. Pre- and post-instrumentation scans were performed using CBCT (Planmeca, Promax 3D max, Helsinki, Finland) to compare transportation resulting from the instrumentation systems.

**Figure 1:**
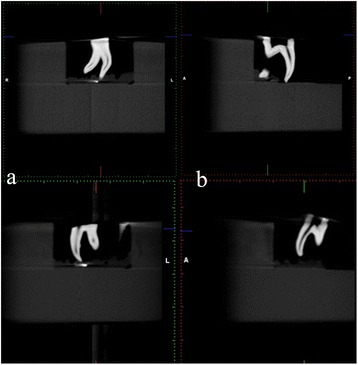
**Teeth were scanned at 96 kVp and 12 mA at two resolutions: (a) 0.125-mm** ^**3**^ **voxel size and (b) 0.100-mm** ^**3**^ **voxel size.**

Scans of each tooth were made at 96 kVp and 12 mA at two resolutions: 0.125 and 0.100 mm^3^ voxel sizes. The field of view was 4.2 cm in diameter and 5.0 cm in height. Slices were 1024 × 1024 pixels. The acquired data were investigated for the following parameters (Figure 1).

### Transportation

**Figure 2:**
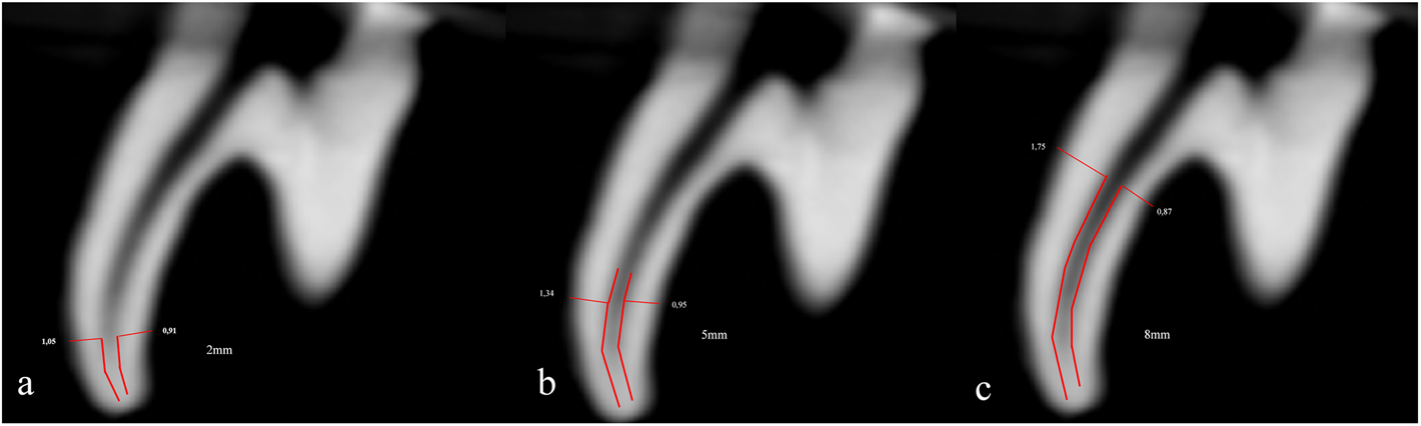
**Pre- and post-instrumented measurements were made in the mesial and distal directions on cross-section planes from the apical end of the root at levels of (a) 2 mm, (b) 5 mm, and (c) 8 mm.**

Three cross-section planes from the apical end of root at levels of 2, 5, and 8 mm were used. The pre- and post-instrumented shortest distances from the edge of the canal to the periphery in all the roots were measured in the mesial and distal directions using the Planmeca software (Romexis ver. 3.2, Planmeca, Helsinki, Finland). Transportation was calculated according to Gambill et al. [25] study. All constructions and measurements were performed on a 21.3-inch flat-panel color-active matrix TFT medical display (NEC MultiSync MD215MG, Munich, Germany) with a resolution of 2048 × 2560 at 75 Hz and 0.17-mm dot pitch, operated at 11.9 bits (Figure 2).

### Canal curvature

**Figure 3:**
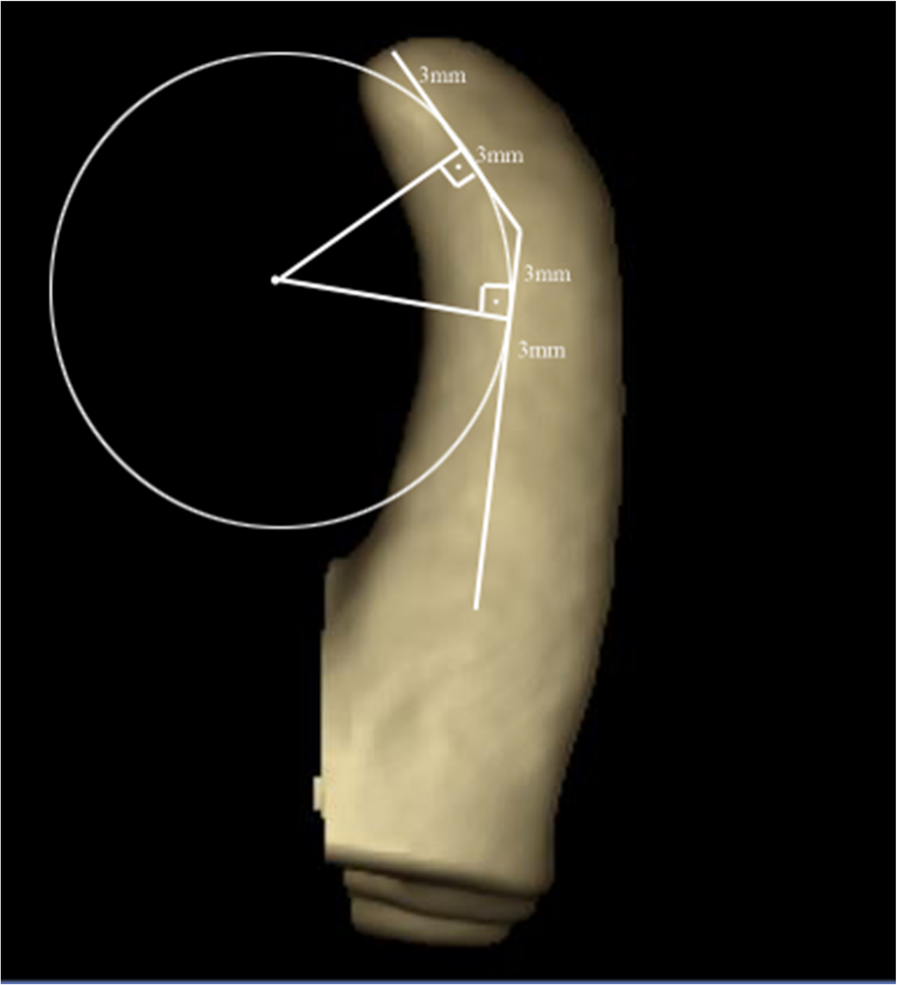
**Three-dimensional CBCT images showing the measurement of root curvature.**

Canal curvature was measured using the 3D Invivo software (ver. 5.1.2., Anatomage, San Jose, CA) following a method described previously [ref.]. Two straight lines of equal lengths were used. The first represented the continuity of the apical region, and the second line followed the middle and coronal thirds of the root canal. The midpoint of each line was determined, and a circle was drawn to pass over the midpoints. The center of the circle was marked, and two lines representing the radii were drawn to the midpoints. The angle between the radii was measured geometrically, and canal curvature was expressed in degrees [1,26]. A curvature radius less than 4 mm (r ≤ 4 mm), considering the two 6-mm semi-straight lines, was classified as severe curvature (25-35°), according to Esterela et al. [1] (Figure 3).

### Volume

**Figure 4:**
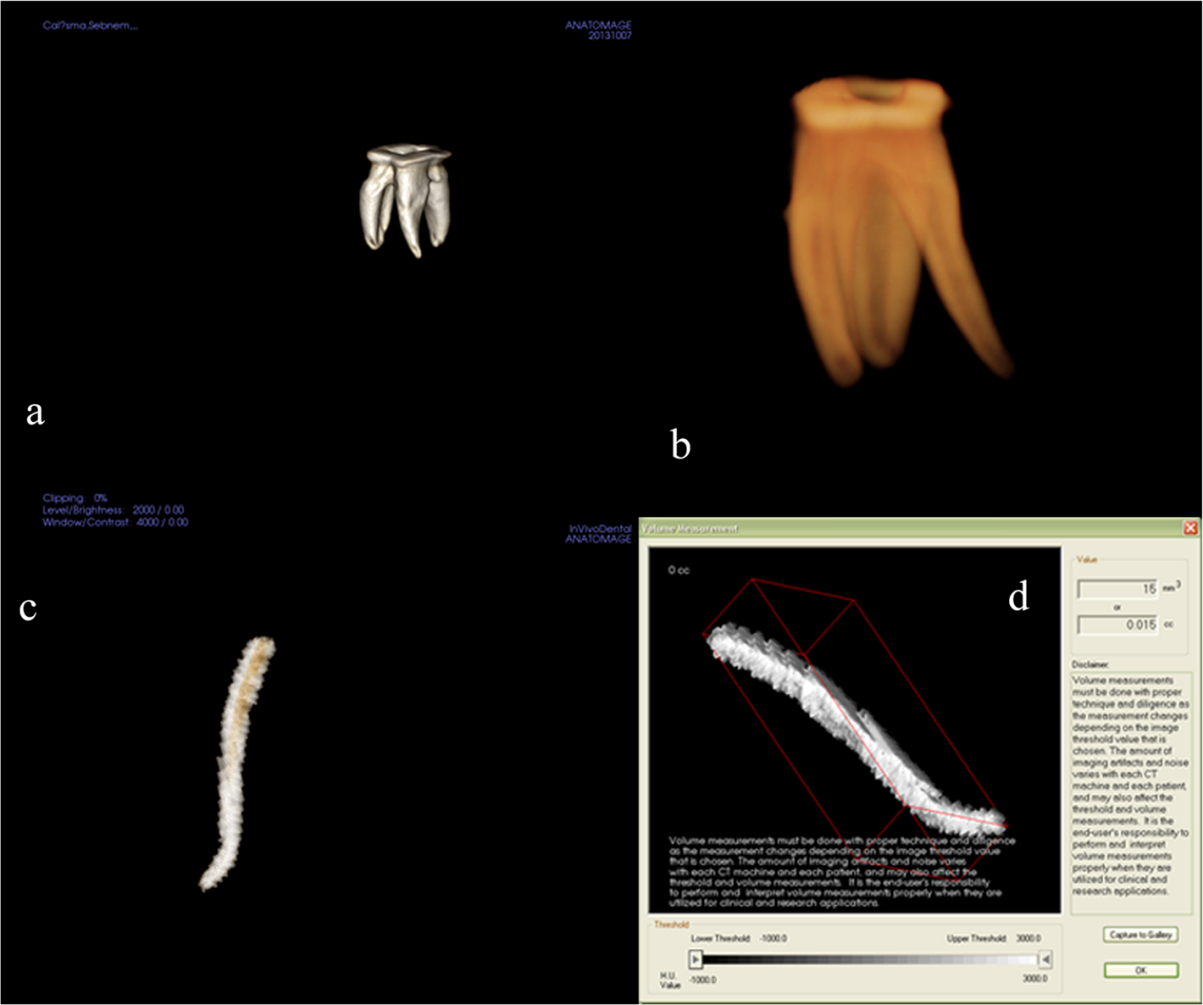
**Three-dimensionally using the 3D Invivo software (ver. 5.1.2., Anatomage, San Jose, CA). a**,**b**. 3D reconstruction of tooth, **c**. subtracted root canal, **d**. The volume of the root canal was measured.

The volume of the mesiobuccal canal was measured before and after instrumentation using the 3D Invivo software. After obtaining axial images from the CBCT data, they were exported in DICOM file format with a 1024 × 1024 matrix and imported into the In-vivo software. 3D surface representations were prepared from the DICOM images. By making the cement and dentin translucent and layering these data, the root canal was observed three-dimensionally (Figure 4). The root canal volume of each tooth was calculated using this software. The software allows the user to “sculpt out” the desired volume from the 3D structure, and, by adjusting the brightness and opacity values, to remove ‘unwanted’ voxels before calculating the final root canal volume.

### Image evaluation

All CBCT images were evaluated retrospectively by two dentomaxillofacial radiologists with 15 years and 7 years of experiences (KO and SK, respectively). The measurements were performed three times at two voxel sizes (0.100- and 0.125-mm^3^) and means of the measurements were recorded as the final measurements. All measurements were taken twice by the same observer, and the mean values of all measurements were included in the statistical analyses. The observers also performed the study twice with an interval of 2 weeks to detect intra-observer variability. Moreover, before starting the radiographic examination in the study, the examiners were calibrated to recognize and identify root anatomy. For this purpose, a set of 10 different CBCT images, not from this study was used. The examiners only examined the CBCTs and were blinded to any other data in the radiographic examination procedure.

### Examiner reliability and statistical analysis

Statistical analyses were performed using the SPSS software (ver. 20.0.1; SPSS, Chicago, IL, USA). Intra- and inter-examiner validation measures were conducted. To assess intra-observer reliability, the Wilcoxon matched-pairs signed-rank test was used for repeated measurements. Inter-observer reliability was determined using the intraclass correlation coefficient (ICC) and the coefficient of variation (CV; CV = (standard deviation / mean) × 100%). Values for the ICC range from 0 to 1. ICC values greater than 0.75 show good reliability, and a low CV demonstrates the precision error as an indicator of reproducibility. Differences according to instrumentation and voxel sizes were made using the Mann–Whitney U-test and the Wilcoxon signed-rank test. Differences were considered significant at *p* < 0.05.

## Results

### Intra-observer consistency

Repeated CBCT measurements indicated no significant intra-observer difference for either observer (*p* > 0.05). Overall intra-observer consistency for Observer 1 was rated at 92% and 94%, while consistency for Observer 2 was 95% and 96% between the two evaluations and measurements, respectively. All measurements were found to be highly reproducible for both observers and there was no significant difference between the two measurements of the observers (p > 0.05).

### Inter-observer consistency

The ICCs between Observers 1 and 2 ranged from 0.89 to 0.91. There was high inter-observer agreement, and the high ICC and low CV demonstrated that the procedure was standardized between the evaluations and measurements of the observers. No significant difference was found among observer evaluations or measurements (p > 0.05). The means of both observers were noted as final measurement data for evaluating canal transportation, curvature, and volumes.

### Canal transportation

Regarding canal transportation, in both the ProTaper Next and New One Shape groups, lower mean transportation values were found at the apical level than at the middle and coronal. A significant difference was found between the apical and coronal levels for both systems (p < 0.05). In comparing the systems, similar values were found at each level with no significant difference (p > 0.05). Moreover, voxel size did not affect the measurements; no significant difference was found between the 0.100- and 0.125-mm^3^ voxel sizes (p > 0.05; Table 1).

**Table 1:** **Mean and standard deviation values of transportation (mm) at different canal levels with two voxel sizes**

**Canal transportation (Level)**	**Voxel size (0.100 mm3)**	**n**	**Mean**	**Standard deviation**	**p value**
2 mm	ProTaper Next	24	0.10^a^	0.09	0.815
	New One Shape	22	0.10^b^	0.08	
5 mm	ProTaper Next	24	0.12	0.08	0.659
	New One Shape	22	0.14	0.09	
8 mm	ProTaper Next	24	0.18^a^	0.10	0.672
	New One Shape	22	0.17^b^	0.09	
**Canal transportation (Level)**	**Voxel size (0.125 mm3)**	**n**	**Mean**	**Standard deviation**	
2 mm	ProTaper Next	24	0.10^c^	0.09	0.572
	New One Shape	22	0.11^d^	0.09	
5 mm	ProTaper Next	24	0.11	0.08	0.778
	New One Shape	22	0.11	0.07	
8 mm	ProTaper Next	24	0.17^c^	0.12	0.625
	New One Shape	22	0.18^d^	0.11	

Same letters indicates statistical significance p < 0.05.

### Canal curvature and volumes

Changes in the pre- and post-instrumented shortest distances from the edge of canal to the periphery in the root were measured in the mesial and distal directions. The results revealed no significant difference between the two systems concerning post-instrumentation canal curvature changes (Table 2). Instrumentation by either of the two tested systems revealed no significant difference in canal volume change (Table 2). Table 3 shows the volumetric and curvature changes according to voxel size. There was also no significant difference in the measurements between the small and large voxel sizes (p > 0.05).

**Table 2:** **Mean and standard deviation values of curvature and removed dentin volume with two voxel sizes**

**Angle of curvature**	**Voxel size (0.125 mm3)**	**n**	**Mean**	**Median**	**Standard deviation**	**p value**
Pre-instrumentation	ProTaper Next	24	24.9	24.0	3.9	0.668
	New One Shape	22	23.2	23.0	2.8	
Post-instrumentation	ProTaper Next	24	22.4	21.2	3.3	0.620
	New One Shape	22	22.1	21.6	2.9	
**Root canal volume**						
Pre-instrumentation (mm^3^)	ProTaper Next	24	9.5	9.0	1.7	0.578
	New One Shape	22	9.5	9.9	1.5	
Post-instrumentation (mm^3^)	ProTaper Next	24	13.2	11.8	2.7	0.421
	New One Shape	22	12.9	11.6	1.9	
**Angle of curvature**	**Voxel size (0.100 mm3)**	**n**	**Mean**	**Median**	**Standard deviation**	**p value**
Pre-instrumentation	ProTaper Next	24	24.6	24.0	3.8	0.518
	New One Shape	22	24.1	23.8	3.2	
Post-instrumentation	ProTaper Next	24	22.8	21.9	3.6	0.648
	New One Shape	22	22.2	21.6	3.0	
**Root canal volume**						
Pre-instrumentation (mm^3^)	ProTaper Next	24	9.6	9.0	1.9	0.528
	New One Shape	22	9.7	10.0	1.6	
Post-instrumentation (mm^3^)	ProTaper Next	24	13.5	12.0	3.1	0.454
	New One Shape	22	12.3	11.5	1.7

**Table 3:** **Mean values of two rotary systems regarding angle curvature and root canal volumes with different voxel values**

**Angle of curvature**	**Resolution**	**n**	**Mean**	**Median**	**Standard deviation**	**p**
Pre-instrumentation	0.100 mm3	23	24.3	23.9	3.5	0.418
	0.125 mm3	23	24.0	23.5	3.4	
Post-instrumentation	0.100 mm3	23	22.5	21.8	3.3	0.798
	0.125 mm3	23	22.3	21.4	3.1	
**Root canal volume**						
Pre-instrumentation (mm^3^)	0.100 mm3	23	9.7	10.0	1.7	0.677
	0.125 mm3	23	9.6	9.0	1.9	
Post-instrumentation (mm^3^)	0.100 mm3	23	13.0	12.0	2.6	0.908
	0.125 mm3	23	12.9	11.0	2.6	

## Discussion

To our knowledge, few reported studies have compared the newly developed rotary systems [12,22-24]. Capar et al. [12] investigated six rotary file systems (ProTaper Next, ProTaper Universal, classical (old) One Shape, Reciproc, Twisted File Adaptive, SM2, and WaveOne) in terms of canal transportation and surface area at 2, 5, and 8 mm above the apex. They used a CBCT system with an 8-cm FOV, 0.075-mm pixel size, and a 0.075-mm slice thickness. They reported no significant difference among the six groups in terms of transportation, canal curvature, change in surface area, or centering ratio after instrumentation. These findings are consistent with the results of the present study.

Consistent with previous studies using similar systems, New One Shape and ProTaper Next showed similar canal transportation. The systems were non-cutting (apical rounded safe tip) systems, leading to minimal apical transportation in curved canals [27]. Another finding of the present study was that the canal transportation values at the 2-mm level were in the range 0.10-0.11 mm. These values are less than the ‘critical’ canal transportation value of 0.3-mm defined by Wu et al. [28].

In the current study, the ProTaper Next and New One Shape instruments respected the original root canal anatomy and behaved similarly, consistent with previous studies [12,22-24]. Bürklein et al. [23] compared Reciproc, WaveOne, HyflexCM, F360, and classical (old) One Shape systems either with or without previous glide path preparation and concluded that less tapered instruments maintained the original canal curvature better than did instruments having greater tapers. Saber et al. [24] compared WaveOne, Reciproc, and the classical (old) One Shape in another study. In that study, the use of One Shape files resulted in significantly greater apical transportation than WaveOne or Reciproc but with no significant difference between WaveOne and Reciproc (P > 0.05). In the mean time Capar et al. [12] evaluated the classical (old) One Shape with five other systems and concluded similar transportation in the preparation of the mesial canals of mandibular molars.

This study showed that the ProTaper Next showed greater volumetric changes in removed dentin than New One Shape, although the difference was not statistically significant. Moreover, according to transportation values, a significant difference was found between the apical and coronal levels for both systems (p < 0.05). It can be interpreted that because the ProTaper Next has less taper in the apical than the coronal regions, canal transportation in the apical regions showed significantly smaller values than the coronal ones. New One Shape has off-centered asymmetrical design like ProTaper Next which similar results were achieved to those with the ProTaper Next. This might be due to the design of the instruments in terms of both having rounded safe tips.

In this study, voxel size changes were also tested. No significant difference was found between the 0.100- and 0.125-mm^3^ voxel sizes. No previous study has attempted to compare voxel sizes for volumetric change and canal transportation, so there are no findings to compare with our results. However, reducing the field of view (FOV) in CBCT images increases the resolution, so more accurate and higher diagnostic capability views are possible [29,30]. Previous studies dealing with root canal geometry have evaluated various voxel sizes in CBCT [31-33]. In a study comparing voxel resolutions (0.125, 0.2, 0.3, and 0.4-mm) in detecting simulated vertical root fractures, no difference was found between voxel sizes. However, accuracy was higher and decisions were easier with 0.125- and 0.2-mm^3^ voxel sizes [34]. Voxel values did not affect the measurements in the present study; no significant difference was found between 0.100- and 0.125-mm^3^ voxel sizes (p > 0.05). Also, in a similar study to detect vertical root fractures, 0.19-, 0.1-, and 0.3-mm voxel sizes were used and the 0.19 and 0.1-mm achieved better resolutions than 0.3-mm, but smaller voxel sizes also mean higher reconstruction times and higher radiation doses [19]. Another study with CBCT scans in horizontal root fractures (HRFs) found the highest accuracy with 0.080- and 0.125-mm^3^ voxel sizes, but with no significant difference. Thus, it was stated that a 0.125-mm^3^ voxel size can be recommended for a flat panel CBCT scanner with good diagnostic performance with a lower exposure dose to detect HRFs [35]. However, further studies should be performed regarding comparisons of larger voxel sizes (0.2, 0.3, and 0.4-mm^3^) versus smaller (0.075, 0.100, and 0.125 mm^3^) voxel sizes.

## Conclusions

ProTaper Next and New One Shape systems produced canal preparations with adequate geometry. The two voxel resolutions also showed similar results. Thus, the ‘best’ voxel resolution would be 0.125 mm because of the shorter scanning time and the reduced radiation exposure for *in vivo* studies.

## Abbreviations

HRF: Horizontal root fracture
CBCT: Cone-beam computed tomography
FOV: Field of view
NiTi: Nickel-titanium
ICC: Intraclass correlation coefficient
NaOCl: Sodium hypochloride
DICOM: Digital imaging and communications in medicine
